# Molecular genetic analysis of FGFR1 signalling reveals distinct roles of MAPK and PLCγ1 activation for self-renewal of adult neural stem cells

**DOI:** 10.1186/1756-6606-2-16

**Published:** 2009-06-08

**Authors:** Dengke K Ma, Karthikeyan Ponnusamy, Mi-Ryoung Song, Guo-li Ming, Hongjun Song

**Affiliations:** 1 Institute for Cell Engineering, Johns Hopkins University School of Medicine, Baltimore, Maryland 21205, USA; 2 Solomon H. Snyder Department of Neuroscience, Johns Hopkins University School of Medicine, Baltimore, Maryland 21205, USA; 3 Department of Biomedical Engineering, Johns Hopkins University School of Medicine, Baltimore, Maryland 21205, USA; 4 Department of Neurology, Johns Hopkins University School of Medicine, Baltimore, Maryland 21205, USA; 5 Bioimaging Research Center and Cell Dynamics Research Center, Gwangju Institute of Science and Technology, Gwangju 500-712, Republic of Korea

## Abstract

**Background:**

Neural stem cells (NSCs) are present in the adult mammalian brain and sustain life-long adult neurogenesis in the dentate gyrus of the hippocampus. In culture, fibroblast growth factor-2 (FGF-2) is sufficient to maintain the self-renewal of adult NSCs derived from the adult rat hippocampus. The underlying signalling mechanism is not fully understood.

**Results:**

In the established adult rat NSC culture, FGF-2 promotes self-renewal by increasing proliferation and inhibiting spontaneous differentiation of adult NSCs, accompanied with activation of MAPK and PLC pathways. Using a molecular genetic approach, we demonstrate that activation of FGF receptor 1 (FGFR1), largely through two key cytoplasmic amino acid residues that are linked to MAPK and PLC activation, suffices to promote adult NSC self-renewal. The canonical MAPK, Erk1/2 activation, is both required and sufficient for the NSC expansion and anti-differentiation effects of FGF-2. In contrast, PLC activation is integral to the maintenance of adult NSC characteristics, including the full capacity for neuronal and oligodendroglial differentiation.

**Conclusion:**

These studies reveal two amino acid residues in FGFR1 with linked downstream intracellular signal transduction pathways that are essential for maintaining adult NSC self-renewal. The findings provide novel insights into the molecular mechanism regulating adult NSC self-renewal, and pose implications for using these cells in potential therapeutic applications.

## Background

Neural stem cells (NSCs) represent a unique type of precursor cells that are capable of self-renewal and differentiation into multiple neural cell types, including neurons and glia [1-3]. During early brain development, NSCs in the germinal region generate numerous progeny in a highly organized manner to construct the nervous system. Adult mammalian brains also harbour a population of adult NSCs that are primarily located in the subventricular zone of the lateral ventricle and the dentate gyrus of the hippocampus to maintain regional ongoing neurogenesis [4-7]. Advances in NSC biology have highlighted the promise of NSCs in stem cell-based therapies for neurological disorders [8-11]. Understanding molecular mechanisms regulating the behaviour of NSCs, including their proper expansion *in vitro *with multipotentiality but not tumorigenicity, is a critical step towards these goals.

As the defining hallmark of stem cells, self-renewal refers to the process by which stem cells expand to generate at least one of the two daughter cells with the same range of developmental potentials as its parental cell [12,13]. Stem cell self-renewal is critical for both embryonic development and adult homeostatic tissue maintenance. In the mammalian brain, NSCs are subject to tight and complex regulation in different regions and at different stages of development. The earliest neuroepithilial NSCs, for example, self-renew and expand rapidly to produce a vast number of progeny in order to meet the need of brain histogenesis. Whereas most adult stem cells *in vivo *usually reside in a micro-environment (niche) and remain relatively quiescent [14], they engage in active self-renewal upon injury signals or under certain physiologic conditions that demand rapid production of new progeny. Due to the complex nature of self-renewal *in vivo*, stem cells in culture provide a better-defined system to investigate how self-renewal is controlled by intrinsic and extrinsic mechanisms.

Emerging evidence suggests that self-renewal is regulated by diverse mechanisms in different stem cells [13,15]. In the case of NSCs, it has long been noted that cell expansion is promoted by the growth factor FGF-2, although little is known about the underlying cytoplasmic signalling mechanism [16-20]. NSCs isolated from different regions of the brain or different stages of development, grown as either "neurosphere" or adherent monolayer culture, all undergo robust proliferation when supplemented with FGF-2 in serum-free defined medium [21-25]. Self-renewal entails not only proliferation but also maintenance of the stem cell state. Cellular sub-cloning experiments showed that the clonal progeny of NSCs still preserved multipotentiality after expansion by FGF-2 [23,26], and *in vitro *expanded adult NSCs retained multipotentiality *in vivo *even after serial transplantation [27]. Genetic ablation of FGF-2 locus in mice resulted in severe defects in the maintenance of a slow-dividing stem cell pool, providing *in vivo *evidence that FGF-2 is necessary for normal NSC self-renewal [28]. Interestingly, FGF-2 is present in normal adult NSC niches, can be induced by diverse types of pathological conditions, and is functionally capable of enhancing the inherently limited self-renewal of endogenous NSCs after ischemic stroke [29-35]. Under different biological contexts, FGF-2 may additionally act in coordination with many other types of extrinsic signalling molecules to exquisitely control adult NSC self-renewal in response to changes of cell physiological milieu, tissue homeostatic states and diverse environmental stimuli [5,10,36-41].

FGF-2 receptors (FGFRs) belong to the family of receptor tyrosine kinases [42,43]. The ligand binding, which is facilitated by heparin, leads to dimerization and autophosphorylation of FGFRs. Consequently, various phosphorylated tyrosine residues on the receptor serve as docking sites for adaptor or enzymatic proteins that link the receptor to downstream intracellular signalling pathways. Previous studies have implicated multiple pathways downstream of FGFRs, including the canonical MAPK (Extracellular signal-regulated kinase, Erk1/2) and phospholipase C (PLC) signalling [42,44]. However, it is unknown whether any of these pathways function in adult NSC self-renewal despite genetic evidence that has clearly implicated the role of FGFR1 in regulating adult NSC proliferation and neurogenesis [32,45,46]. Erk1/2 activation, for instance, has been shown to be important for myoblast proliferation, whereas its suppression promotes self-renewal of mouse embryonic stem cells [47,48]. These findings suggest that signalling pathways are largely conserved, yet their effects are context-dependent [42]. Thus, it is necessary to analyze the specific role of a given pathway in a particular cellular process.

In this study, we aim to gain molecular understanding on the role and mechanism of FGFR signalling in regulation of adult NSC self-renewal. Choosing the well-established rat hippocampal adult NSCs as our model system, we undertook multiple experimental strategies to assess whether specific FGFR signalling is sufficient to promote the self-renewal of adult NSCs, and further dissect out the functional requirement and cooperation of MAPK, PLC pathways in FGF-2-dependent self-renewal of adult NSCs.

## Results and discussion

### FGF-2 regulates the self-renewal of adult NSCs through promoting proliferation and inhibiting spontaneous differentiation

**Figure 1:**
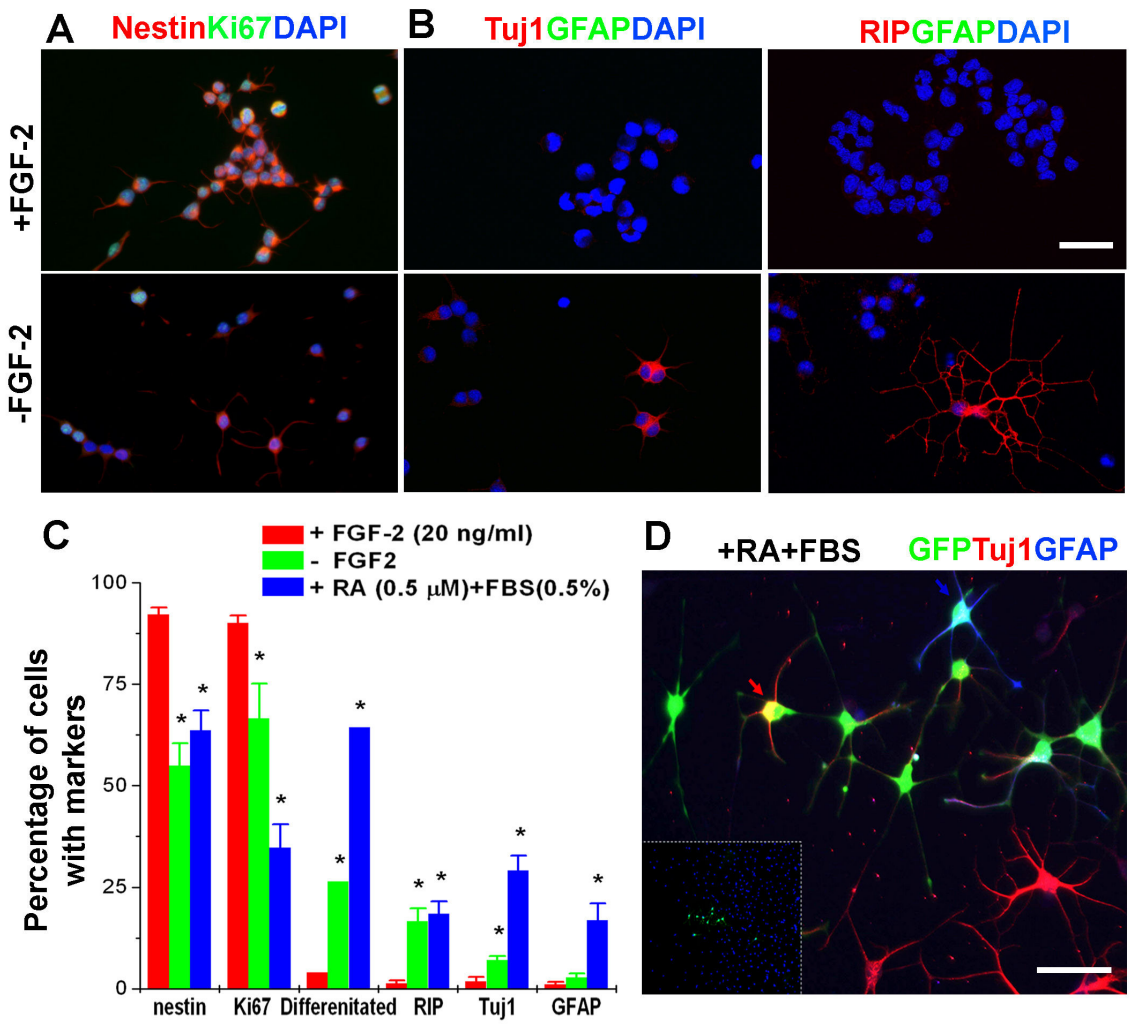
**FGF-2 regulates self-renewal of adult NSCs by promoting proliferation and inhibiting spontaneous differentiation**. **(A, B)** Sample immunostaining images of adult NSC culture with or without treatment of exogenous FGF-2 (20 ng/ml). Nestin is a neural precursor cell marker; Ki67 is a cell proliferation marker; Tuj1 is a neuronal marker; RIP is an oligodendrocyte marker; GFAP is an astrocyte marker. Scale bar: 20 μm. **(C)** Quantification of the percentage of cells with characteristic markers in the presence or absence of FGF-2, or after treatment of RA (0.5 μM) and FBS (0.5%). Values represent mean ± SEM. (n = 6; *: P < 0.01, Student's t-test). **(D)** Multi-lineage differentiation potentials of adult NSCs after long-term culture in the presence of FGF-2. EGFP was used to label a single cell and allowed to expand in the presence of FGF-2 (20 ng/ml) and then induced to differentiate into Tuj1^+ ^neurons (red) and GFAP^+ ^glia (blue) with RA (0.5 μM) and FBS (0.5%) for 6 days. Scale bar: 20 μm.

When grown as monolayer cultures, adult rat hippocampal NSCs remain multipotent and their self-renewal is strictly dependent on FGF-2 (Additional file 1). Initially isolated and purified from adult rat hippocampus, these adult NSCs can be maintained for long-term in serum-free F12/N2 medium supplemented with 20 ng/ml FGF-2 [22,24,25,49]. They give rise to neurons, astrocytes and oligodendrocytes both in culture and after transplanted into the dentate gyrus of adult rats *in vivo *[49,50]. Clonal-derived adult NSCs retain multi-lineage potentials, consistent with an FGF-2-dependent self-renewal of adult NSCs (Figure 1). To evaluate the effect of FGF-2 on adult NSC self-renewal, we assessed several integral aspects of stem cell self-renewal: proliferation, anti-differentiation, and maintenance of multipotentiality.

In the presence of FGF-2, the adult NSC culture comprised mostly Nestin^+ ^(a neural progenitor maker) and Ki67^+ ^(a proliferation marker) population (Figure 1A). Differentiation markers Tuj1 (neuronal), GFAP (astroglial), and RIP (oligodendroglial) were rarely detected (Figure 1B). By contrast, withdrawal of FGF-2 led to significant cell cycle arrest and spontaneous differentiation within 4 days, as shown by a significant decrease in the percentage of Ki67^+ ^and Nestin^+ ^cells and an increase of spontaneous neuronal and oligodendroglial differentiation (Figure 1B, C). Overall, the percentages of apoptotic cells were not significantly altered with or without FGF-2 under these culture conditions. When the multipotentiality of adult NSCs was examined at different passages (passage 15, 25, 35), the culture consistently generated both neurons and glia. Furthermore, EGFP-labelled clonal-derived adult NSCs gave rise to both Tuj1^+ ^neurons and GFAP^+ ^astrocytes (Figure 1D). These results suggest that FGF-2 promotes self-renewal of NSCs by stimulating proliferation, inhibiting spontaneous differentiation, and maintaining multipotentiality.

### A chimeric receptor recapitulates effects of FGF-2 and implicates Erk1/2 and PLCγ1 signalling in adult NSC self-renewal

**Figure 2:**
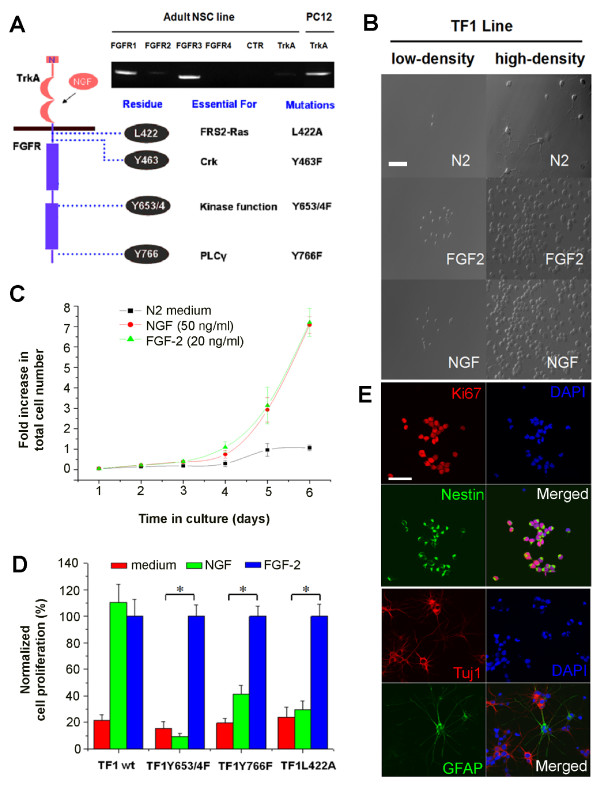
**A chimeric receptor recapitulates effects of FGF-2 and implicates Erk1/2 and PLCγ1 signalling in adult NSC self-renewal**. **(A) **A schematic diagram illustrating the chimeric receptor and the amino acid residues within the FGFR1 intracellular domain that are linked to various downstream signalling pathways. Shown on the top is the RT-PCR analysis of the expression of endogenous TrkA and FGFRs under the proliferating condition in the presence of FGF-2. **(B) **A bright-field view (low and high density) of NSC lines with the TF1 chimeric receptor grown in the presence of FGF-2 (20 ng/ml) or the surrogate ligand NGF (50 ng/ml). Scale bar: 20 μm. **(C) **Growth curves of the NSC lines with TF1 chimeric receptors in the absence, and presence of FGF-2 (20 ng/ml) or the surrogate ligand NGF (50 ng/ml). Values represent mean ± SEM. (n = 3). **(D) **Quantification of NSC numbers of various chimeric receptor lines cultured in the medium alone, or with supplementation of FGF-2 (20 ng/ml) or NGF (50 ng/ml). For each line, the total NSC numbers were normalized to those cultures in the presence of FGF-2 (20 ng/ml). Values represent mean ± SEM. (n = 3; *: P < 0.01, Student's t-test). **(E) **Normal NSC characteristics and multipotentiality of the TF1 chimeric NSC line maintained by NGF. Shown on the top are sample images of immunostaining of proliferating NSCs with Ki67 (red) and Nestin (green). Shown on the bottom are sample images of immunostaining with Tuj1 (red) or GFAP (green) of cultures at 6 days after the treatment of RA (0.5 μM) and FBS (0.5%). Scale bar: 20 μm.

How does FGF-2 exert such wide-ranging effects on adult NSCs? Among the four members of FGFRs, FGFR1 was highly expressed in adult NSCs (Figure 2A). These adult NSCs exhibited little endogenous NGF receptor TrkA transcript during proliferation (Figure 2A). To test whether FGFR1 activation is sufficient to promote self-renewal, we derived an adult NSC line harbouring a chimeric receptor with the extracellular domain of NGF receptor TrkA and the intracellular domain of FGFR1 (TF1 line; Figure 2A). In the chimeric NSC line, NGF was sufficient to activate FGFR1 signalling and mimic effects of FGF-2 in promoting long-term proliferation and inhibiting differentiation of adult NSCs (Figure 2B, C). Importantly, the chimeric TF1 NSC line remained to be responsive to FGF-2, and multipotent after long-term culture in the present of NGF (Figure 2B, C, E), suggesting that FGFR1 signalling is sufficient to promote proliferation and maintain multipotentiality of adult NSCs.

By expressing a chimeric TrkA-FGFR receptor, we used NGF as a surrogate ligand to activate FGFR1 and examined the influence of specific mutations from the intracellular domain of FGFR1 on adult NSC self-renewal. We established lines of adult NSCs with a series of chimeric receptor constructs, including TrkA-FGFR1 (TF1), TF1L422A, TF1Y463F, TF1Y653/4F, and TF1Y766F (Figure 2A). L422 is a critical leucine amino acid residue site for FRS2 binding, and its mutation leads to loss of downstream signalling through the FRS2-Ras-MAPK cascade [51]. Y653/4F (tyrosine to phenylalanine) is an FGFR1 kinase enzymatic inactive mutation, and Y463F and Y766F disrupt substrate actions of the tyrosine kinase Crk and a member of the PLC family, PLCγ1, respectively [44,52]. In NSC proliferation assay, NGF recapitulated the effect of FGF-2 for the TF1 line, whereas NGF failed to stimulate the proliferation of the TF1Y653/4F kinase dead mutant line (Figure 2D). In contrast, NGF-induced expansion of TF1L442A, TF1Y766F lines were significantly decreased compared to the TF1 line. Importantly, all these adult NSC lines retained normal self-renewal in response to FGF-2 (Figure 2D). Taken together, these results indicate that L442 and Y766 linked downstream Ras-MAPK and PLCγ1 activation are likely essential for maintaining adult NSCs, through direct regulation of NSC proliferation and/or maintenance of progenitor characteristics.

**Figure 3:**
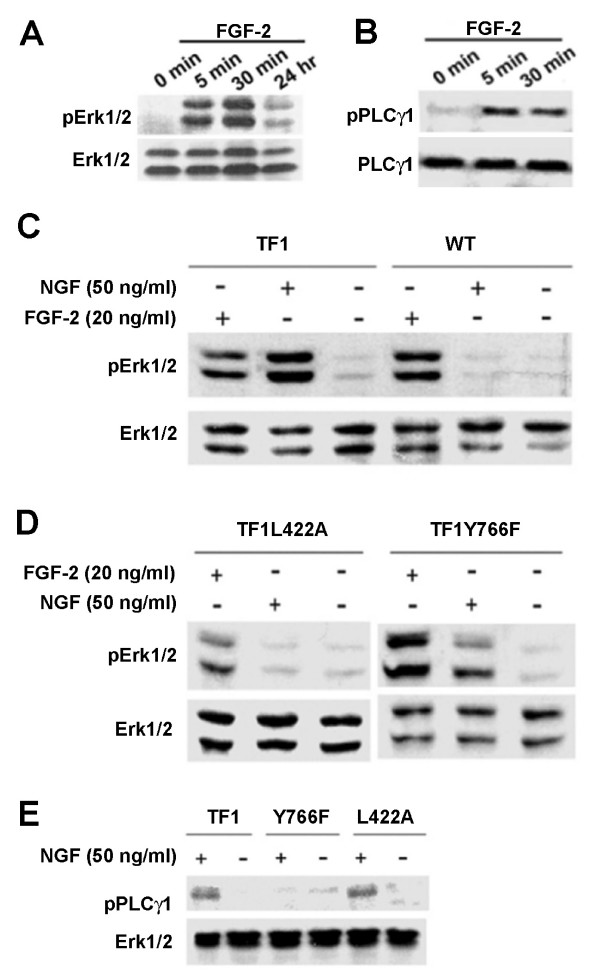
**Specific activation of signal transduction pathways in chimeric receptor lines with various mutations of several key amino acid residues of FGFR1**. (**A-B**) Western blot analysis of Erk1/2 and PLCγ1 phosphorylation in normal adult NSCs in response to FGF-2 (20 ng/ml). (**C-D**) Western blot analysis of NGF-induced Erk1/2 phosphorylation in the TF1 line and in the mutant TF1L422A or TF1Y766F chimeric lines. (**E**) Western blot analysis of NGF-induced PLCγ1 phosphorylation in the mutant TF1L422A or TF1Y766F chimeric lines.

Using phospho-specific antibodies against Erk1/2 and PLCγ1, western blot analysis showed that FGF-2 induced prominent Erk1/2 and PLCγ1 activation (Figure 3A). While Erk1/2 phosphorylation persisted into 24 hours after the addition of FGF-2, PLCγ1 tyrosine phosphorylation appeared to be transient in nature. The dependence of Erk1/2 and PLCγ1 activation on L442 and Y766 residues was confirmed in chimeric NSC lines with respective signalling deficiencies (Figure 3C to 3E). Collectively, these results suggest that two key amino acid residues in the intracellular domain of FGFR1 are important for adult NSC self-renewal and mediate the effects of FGF-2 through ERK and PLCγ1 signal transduction pathways.

### Activation of Erk1/2 is both required and sufficient for the proliferation of adult NSCs

**Figure 4:**
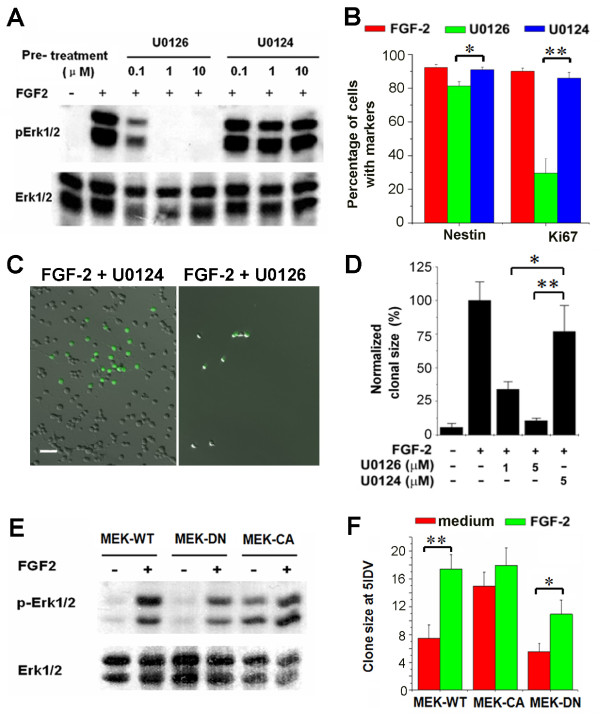
**Erk1/2 Activation is both required and sufficient for adult NSC proliferation**. **(A) **Western blot analysis of Erk1/2 inhibition in normal adult NSCs with the treatment of U0126 or U0124 at different concentrations. **(B) **Quantification of the cellular composition of adult NSC culture in the presence or absence of U0126, or its inactive analog U0124 (2.5 μM). Values represent mean ± SEM. (n = 6; *: P < 0.01, Student's t-test). **(C) **Sample images of clonal analysis of adult NSCs with MEK1 inhibition. Scale bar: 20 μm. **(D) **Summary of clonal analysis of adult NSCs in the presence of U0126 or U0124. **(E) **Western blot analysis of Erk1/2 activation in MEK1-WT, DN or CA lines. **(F) **Summary of clonal analysis assay for adult NSCs expressing MEK1-DN, WT or CA. Values represent mean ± SEM. (n = 3; *: P < 0.01, Student's t-test).

To directly examine the specific role of Erk1/2 activation in adult NSC self-renewal, we treated adult NSC cultures with U0126, a selective and potent inhibitor for the Erk1/2 kinase MEK1/2 [53]. As shown by western blot analysis (Figure 4A), FGF-2-stimulated Erk1/2 activation was inhibited by U0126 in a dose-dependent manner. In adult NSC culture treated with 2.5 μM U0126, the percentage of Ki67 or Nestin positive cells was significantly lower than the untreated culture (Figure 4B). In contrast, U0124, the inactive analogue of U0126, elicited no significant effects. When subjected to clonal analysis assay in measuring self-renewal expansion at the single cell level (Additional file 2), U0126 also suppressed FGF-2-induced clonal expansion of EGFP-labelled NSCs in a dose-dependent manner (Figure 4C, D).

To further examine the role of Erk1/2 activation in adult NSC proliferation, we engineered retroviruses to over-express the dominant negative (DN), wild-type (WT) and constitutively active (CA) mutants of MEK1 in adult NSCs (Figure 4E, F). These mutants have been widely used to manipulate cellular Erk1/2 activity [54,55]. Bicistronic expression of EGFP was used to monitor transduced cells with infection efficiency over 95%. Western blot analysis using phosph-Erk1/2 antibodies confirmed that MEK1-DN NSCs effectively attenuated Erk1/2 activation, and MEK1-CA rendered Erk1/2 constitutively active in adult NSC culture (Figure 4E). In clonal analysis assay, MEK1-DN NSCs produced significantly reduced clone sizes in the FGF-2 treated condition, whereas MEK1-CA NSCs yielded significantly increased clone sizes even in the absence of FGF-2 (Figure 4F). Collectively, these data suggest that Erk1/2 activation is both required and sufficient for FGF-2-dependent proliferation of adult NSC.

### Activation of Erk1/2 blocks both spontaneous and induced differentiation of adult NSCs

**Figure 5:**
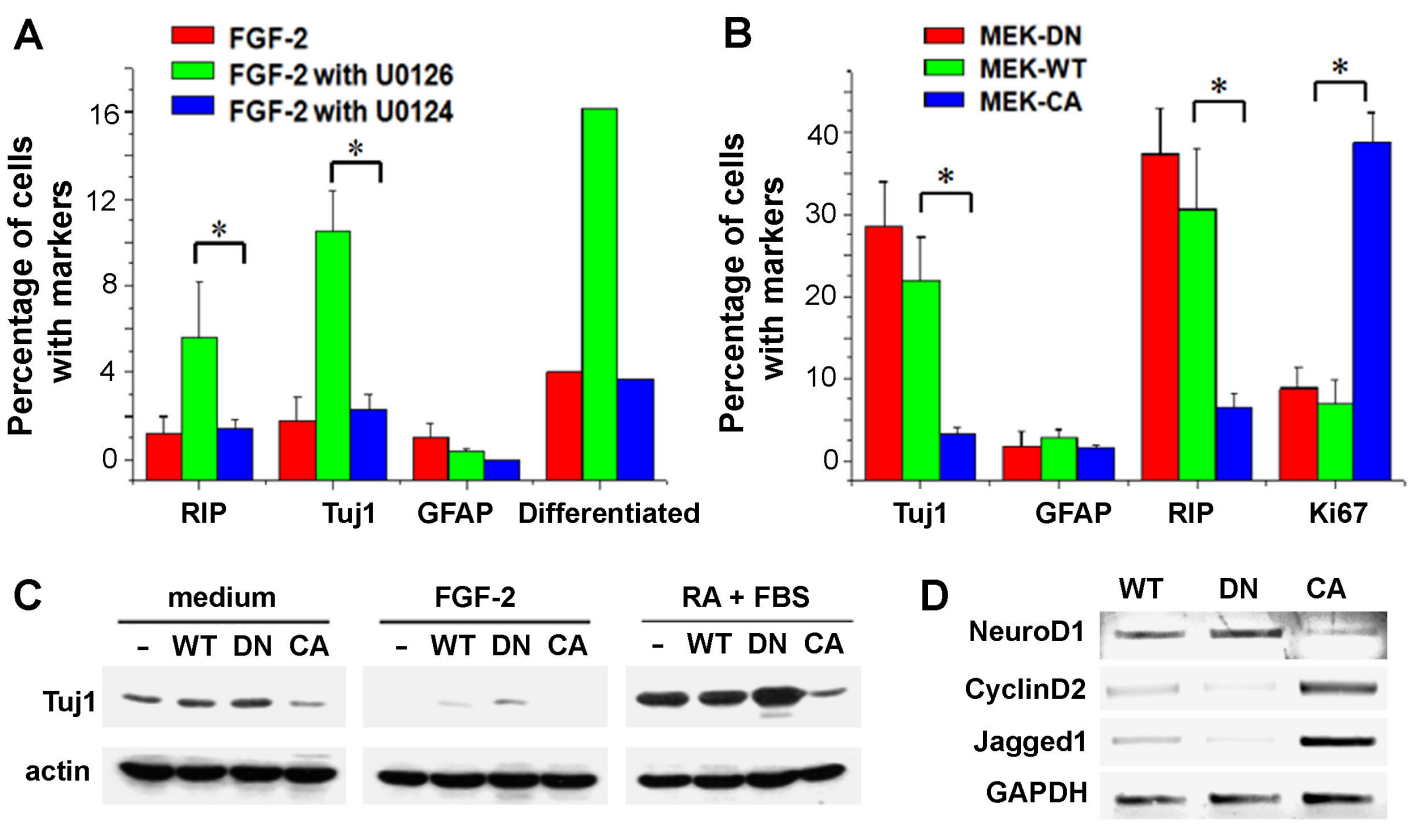
**Erk1/2 Activation blocks both spontaneous and induced differentiation**. **(A) **Cell number quantification for each differentiation marker (Tuj1, GFAP or RIP) in proliferating adult NSC culture in the presence of FGF-2 (20 ng/ml) and with U0126 or U0124 (2.5 μM) (n = 6; *: P < 0.01, Student's t-test). **(B) **Cell number quantification for each differentiation marker (Tuj1, GFAP or RIP) in adult NSCs expressing MEK1-WT, DN, or CA at 6 days after the induction of differentiation with RA (0.5 μM) and FBS (0.5%) (n = 3; *: P < 0.01, Student's t-test). **(C) **Western blot analysis of neuronal marker Tuj1 expression in adult NSCs expressing MEK1-WT, DN, or CA under the medium only, FGF-2 supplemented and normal differentiation with RA (0.5 μM) and FBS (0.5%) conditions. **(D) **Semi-quantitative RT-PCR analysis on the induction of NeuroD, CyclinD2 and Jag1 genes in adult NSCs expressing MEK1-WT, DN, or CA under the differentiation condition.

Spontaneous differentiation of some adult NSCs was also observed after U0126 treatment or MEK1-DN expression. Next, we examined the role of Erk1/2 pathway in the anti-neuronal differentiation effect of FGF-2 on adult NSCs in detail. Spontaneous NSC differentiation occurred at a very low basal level in the presence of FGF-2 (Figure 5A). Treatment of U0126 (2.5 μM), but not U0124, led to significant spontaneous differentiation of adult NSCs into RIP^+ ^oligodendrocytes and Tuj1^+ ^neurons (Figure 5A). Similarly, the NSC line expressing MEK1-DN exhibited a higher spontaneous differentiation rate than both MEK1-WT and MEK1-CA lines. In standard differentiation condition with the treatment of retinoic acid (RA; 0.5 μM) and fetal bovine serum (FBS; 0.5%) for 6 days [24,56], adult NSC lines expressing MEK1-CA generated a significantly lower percentage of RIP^+^oligodendrocytes and Tuj1^+ ^neurons (Figure 5B). Meanwhile, most cells from MEK1-CA NSC lines remained in cell cycle as indicated by the significantly higher percentage of Ki67^+ ^cells (Figure 5B). We further explored these changes in neuronal differentiation by performing western blot analysis. Tuj1 was up-regulated in the absence of FGF-2 and reached a higher level under differentiation conditions (Figure 5C). Such increase was accelerated in the MEK1-DN NSC lines and abrogated in the MEK1-CA NSC lines.

Considering the phenotype of MEK1 NSC lines, we also tested whether expression and regulation of key genes involved in proliferation and differentiation were affected in MEK1 NSC lines. Indeed, NeuroD1, an essential transcription factor for neuronal differentiation, was strongly down-regulated in the MEK1-CA NSC line compared to the MEK1-DN and WT NSC lines (Figure 5D). Interestingly, CyclinD2, one of the key genes for cell cycle progression showed a reverse expression pattern as NeuroD1. It has been shown that FGF and Shh control CyclinD2 and CyclinD1 expression to maintain the cycling and undifferentiated progenitor states at different brain regions with NSCs, respectively [57]. While it is likely that proliferation and anti-differentiation are coupled events, Erk1/2 may also promote cell cycle progression and inhibit precocious differentiation programs by independent mechanisms. One of the prominent targets is Notch signalling, which has been shown to inhibit neuronal differentiation from multiple types of NSCs and its constitutive activation led to astrocyte differentiation [58]. In adult NSCs, we detected abundant expression of a Notch ligand Jagged-1 in the MEK1-CA, but not in MEK1-DN or WT NSCs, suggesting that Jagged-1 and Notch signalling may mediate the anti-differentiation effects of MEK1 (Figure 5D). Taken together, these results indicate that the MAPK-ERK pathway of FGFR1 signalling prevents both spontaneous and induced neuronal and oligodendroglial differentiation, possibly through regulation of key genes including NeuroD1 and CyclinD2.

### PLCγ1 maintains neuronal and oligodendroglial differentiation potentials of adult NSCs

**Figure 6:**
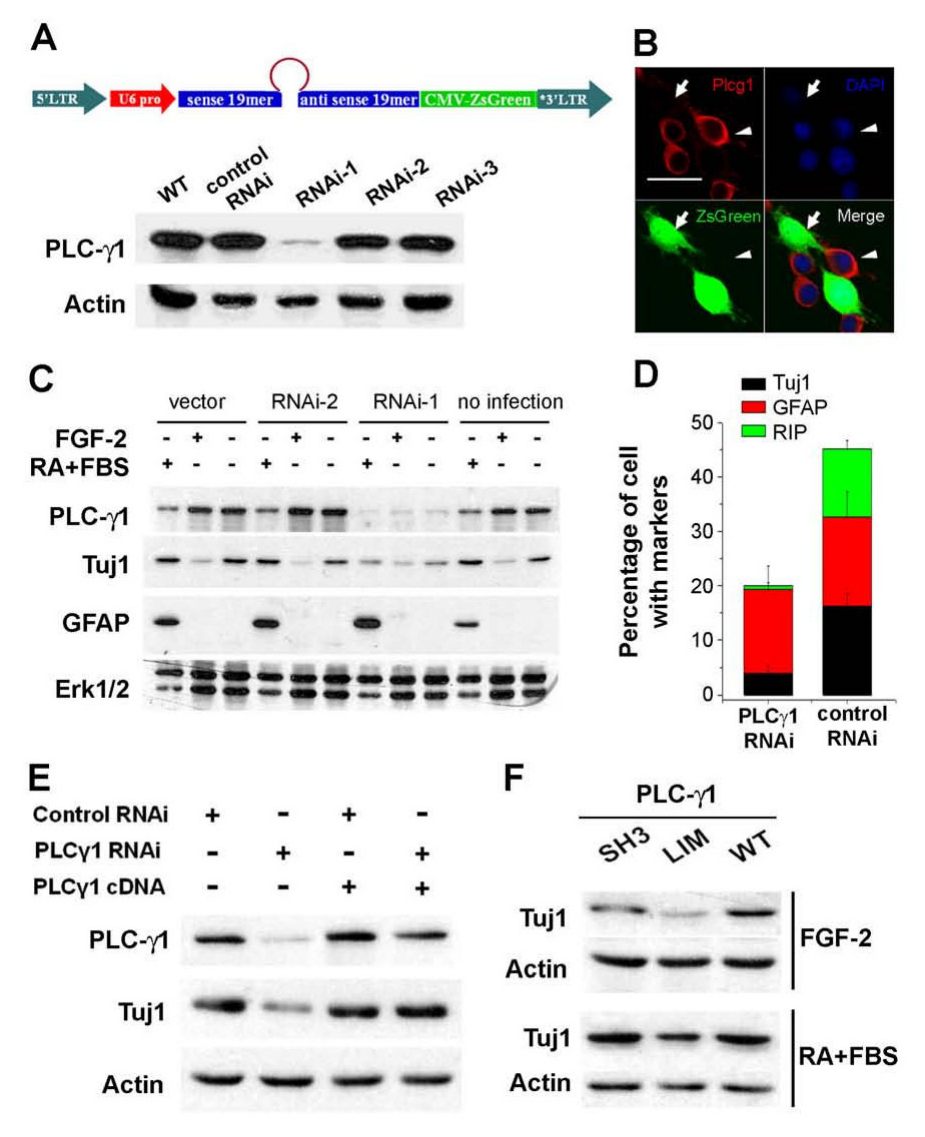
**Impairment of neuronal and oligodendrocyte differentiation in PLCγ1-depleted adult NSCs**. **(A) **A schematic diagram illustrating the retroviral shRNA construct and western blot analysis on the efficacy of knockdown by shRNAs against endogenous PLCγ1 in adult NSCs. **(B) **Immunostaining of endogenous PLCγ1 in NSCs infected with shRNA-i1. Scale bar: 20 μm. **(C) **Western blot analysis of neuronal differentiation marker Tuj1 and glial marker GFAP in PLCγ-depleted NSCs in the presence or absence of FGF-2, or after induction of differentiation with RA (0.5 μM) and FBS (0.5%) for 6 days. **(D) **Quantification of various cell types in PLCγ1-depleted NSCs after induction of differentiation with RA (0.5 μM) and FBS (0.5%) for 6 days. Values represent mean ± SEM. (n = 4; *: P < 0.01, Student's t-test). **(E) **Rescue of neuronal differentiation defects of endogenous PLCγ1-depleted adult NSCs by exogenous expression of PLCγ1 cDNA plasmid. **(F) **Western blot analysis of neuronal differentiation marker Tuj1 after differentiation of adult NSCs expressing WT and two different dominant negative mutants of PLCγ1 (LIM: lipase inactive mutant, SH3: SH3 domain deletion mutant).

In parallel with the MAPK pathway, we also examined how PLCγ1 might participate in regulating adult NSC self-renewal. To directly ascertain the function of PLCγ1, we designed and screened a number of small hairpin RNAs (shRNA) to knockdown endogenous PLCγ1 expression in adult NSCs (Figure 6A). Retroviruses carrying the shRNAs along with a visualizing marker ZsGreen were used to infect adult NSCs. Both western blot analysis and immunostaining confirmed the knockdown efficacy of one shRNA from the screen (Figure 6A–B). This shRNA targets the 3'UTR region to allow rescue experiments with the exogenous full-length cDNA of PLCγ1.

Under normal proliferation conditions, PLCγ1-depleted cells exhibited decreased number of GFAP negative and Nestin positive cells, consistent with results from the mutant chimeric receptor NSC line TF1Y766F (Figure 2D). We next assessed the differentiation profile of adult NSCs infected with PLCγ1-shRNA and control shRNA viruses. Surprisingly, in the standard differentiation condition with 0.5 μM RA and 0.5% FBS for 6 days, the majority of the progeny of PLCγ1-shRNA cells consisted of GFAP^+ ^astrocytes, instead of a mixed population of neurons and glia as seen in control (Figure 6D). Cell death rates were not significantly altered in these conditions. A large fraction of PLCγ1-depleted cells remained to be undifferentiated even with RA and FBS (Figure 6D), in sharp contrast to adult NSCs with Erk1/2 inhibition, which led to reduced proliferation and elevated neuronal differentiation of adult NSCs (Figure 5). Even in the normal proliferation condition, the number of GFAP positive and Nestin negative cells slightly increased among PLCγ1-depleted cells. Results from immunostaining-based quantitative cell counting were further supported by western blot analysis (Figure 6C and Additional file 3). The amounts of neuronal and oligodendroglial differentiation markers Tuj1 and CNPase, but not astroglial marker GFAP, were significantly less abundant in PLCγ1-shRNA cells than the normal, vector transduced, or control shRNA virus transduced adult NSCs after induction of differentiation. Importantly, introducing WT PLCγ1 cDNA back into the PLCγ1-shRNA cells rescued the impaired neuronal differentiation capacity of cells depleted with PLCγ1, excluding potential off-target effects of this particular shRNA (Figure 6E). Furthermore, adult NSCs expressing the lipase-inactive DN form of PLCγ1 [59] exhibited similar deficits of neuronal differentiation (Figure 6F). These experiments demonstrate that knockdown of PLCγ1 leads to largely exclusive commitment of adult NSC towards astroglial fates, suggesting an essential role of PLCγ1 to maintain the full developmental potential of adult NSCs for proper neuronal and oligodendroglial differentiation.

Intriguingly, the phenotype of PLCγ1-depleted cells resembles glioblastoma, a type of brain tumor cells that also exhibit impaired capacity for neuronal and oligodendroglial differentiation [60,61]. Accumulating evidence also suggests that adult NSCs *in vivo *express GFAP, an astrocytic marker, and glioblastoma may originate from adult NSCs [60,61]. In addition to the impaired neuronal and oligodendroglial differentiation, PLCγ1-shRNA cells exhibited enhanced GFAP expression in the presence of FGF-2 (Figure 6C). It is thus plausible that PLCγ1 normally regulates the transition of multipotent NSCs into astrocyte versus other fates, and its depletion may predispose NSCs to glial differentiation thus compromising multipotentiality. Consistent with this notion, PLCγ1 is abundantly expressed by embryonic radial glia during fetal brain development, and its overall expression is inversely correlated with GFAP expression from the embryonic stage E14 to adulthood [62].

Our results support a model that FGF-2 induced Erk1/2 activation promotes the proliferation and blocks the spontaneous neuronal and oligodendrocyte differentiation of adult NSCs, while in parallel FGF-2-induced activation of PLCγ1 pathway maintains the full differentiation capacity of NSCs into neuronal and oligodendroglial lineages by preventing excess astroglial commitment of adult NSCs (Additional file 4). Other pathways downstream of FGF-2 or alternative signal transduction machineries, such as EGF, BMP, WNT, SHH, and cytokine signalling molecules, may also interact with the pathways studied in our work, and converge on the regulation of adult NSC self-renewal in a context-specific and coordinated manner [36,38,40,46,63-70]. Given that FGF-2 is normally expressed in adult NSC niches, induced by diverse injuries such as ischemic stroke, and promotes the mobilization and self-renewal of adult NSCs in certain physiological and pathologic conditions [29-35], it will be of interest in the future to investigate the involvement and functionality of these FGF-2 dependent intracellular signalling pathways in regulating adult NSC self-renewal *in vivo *in normal and disease contexts.

## Conclusion

Understanding molecular mechanisms of stem cell behaviour regulated by extrinsic factors is an important step towards therapeutic application of NSCs for neurodegenerative diseases. Here we identified two key intracellular signalling pathways that dictate distinct aspects of adult NSC self-renewal in response to FGF-2. Erk1/2 pathway mediates both the proliferation and anti-neuronal differentiation effects of FGF-2, whereas PLCγ1 maintains adult NSC characteristics and developmental potentials of adult NSCs for neuronal and oligodendroglial differentiation. Coordination of these two pathways ensures that adult NSC self-renewal is under the stringent control of growth factor signalling, and to potentially prevent adult NSCs from transforming into cancerous stem cells such as glioblastoma, and losing precocious multipotentiality.

FGF-2 signaling is essential for self-renewal of adult neural stem cells from multiple mammalian species, including humans (Additional file 1). Our findings provide mechanistic insights into the molecular and cellular machinery regulating adult NSC self-renewal. Molecular genetic dissection of the FGFR1 pathway in this study also suggests novel biomarkers and interventions for monitoring and preserving desired NSC states, and thus have clear implications for potential uses of adult NSCs expanded *in vitro *in therapeutic applications.

## Methods

### Isolation, Culturing and Differentiation of Adult NSCs

The adult NSC line was initially established from primary adult rat NSCs [22,24]. These adult NSCs were isolated from hippocampi of adult (3-month-old) male Fischer 344 rats. Briefly, hippocampi were dissected and transferred to PBS medium containing penicillin and streptomycin. Tissue was washed, minced, and enzymatically digested for about 30 min in a mixture of 0.1% neural protease, 0.01% papain and 0.01% DNAse I. Tissue was then mechanically dissociated and cells were washed, centrifuged, and resuspended in DMEM containing 10% FBS. Equal volume of Percoll was added, and cells were centrifuged at 12,700 RPM for 30 min. The middle layer of the gradient were removed and washed 3 times with PBS. Cells were then resuspended and counted before plated on laminin-coated flasks in DEME/F12 medium containing N2 supplement, L-glutamine (2 mM) and FGF-2 (20 ng/ml) as described [22,24]. Cells were passaged for expansion when reaching 70% confluence or seeded at clonal density for experiments. For differentiation studies, fresh RA (0.5 μM) and FBS (0.5%) were added to FGF-2 free culture for six days and the medium was changed every 3 days with fresh RA and FBS.

### Constructs and molecular biology

The original chimeric TF1 constructs [71] were sub-cloned into the retroviral vector pBMN-IRES-EGFP upstream of IRES-EGFP. Mutagenesis was performed by QuickChange (Stratagene) and confirmed by sequencing. The vector pSilencer-RetroQ (Clontech) was used to amplify the fragment containing the U6 promoter by a universal sense primer and an shRNA-containing antisense primer. PCR products were cloned into pSilencer-RetroQ to generate retroviral vectors. The primer sequence for PLCγ1 shRNA was as follows: PLCγ1: 5'-CTAGAATTCACGCGTAAAAAAGAAACAACCGGCTCTTCGTCCAAGCTTCGACGAAGAGCCGGTTGTTTCGGATCCTCGTCCTTTCCACA. Scrambled sequences were used as controls.

### Virus Production and Transduction

Phoenix Eco-packaging cell line or 293-gp cells (Clontech) were transfected with retroviral vectors pseudotyped with VSVG by calcium phosphate methods as previously described [72,73]. Supernatant was collected and subject to ultra-centrifugation (25 k rpm, 90 min). Titer of virus was determined in NIH3T3 cells and aliquots were frozen at -80°C. Transduction was performed overnight with 2 μg/ml polybrene in a minimum volume of medium as previously described [74].

### Immunocytochemistry and in Vitro Quantification

Cells were fixed with 4% paraformaldehyde, followed by immunocytochemical staining as described [22,24,56]. The following primary antibodies were used: rabbit anti-Tuj1 (1:7500; Covance), mouse anti-RIP (1:50; Hybridoma Bank); guinea pig anti-glial fibrillary acidic protein (GFAP; 1:2,500; Advanced Immunochemical), mouse anti-PLCγ1 (1:1000; Upstate). After incubation with secondary antibodies (1:250; Jackson Immunoresearch) for 90 min at room temperature, cultures were rinsed, stained with 4',6-diamidino-2-phenylindole (DAPI), rinsed, mounted and stored at 4°C. Images were taken with fluorescence confocal microscopy system (Zeiss LSM510). All experiments were independently replicated at least three times.

### Clonal analysis assay

Retrovirus transduced NSCs were mixed with WT NSCs at a clonal ratio (generally 1:40) and assayed for clone size and clonal composition in various conditions. For each chimeric receptor, infected culture was grown in the presence of FGF-2 (20 ng/ml), NGF (50 ng/ml), or medium only. After 4 days, the size of EGFP clones was counted and quantified; cell death events in each clone was assessed alive by propium iodide and Hoescht staining; differentiation states was examined by immunostaining with Nestin, Tuj1, RIP and GFAP. Another duplicate set of cultures was allowed to differentiate in the presence of RA (0.5 μM) and FBS (0.5%) for six days and the clonal composition (Tuj1^+^/RIP^+^/GFAP^+^) was examined for multipotentiality.

## Competing interests

The authors declare that they have no competing interests.

## Authors' contributions

DKM and KP: conception and design, collection of data, data analysis, manuscript writing; MS: original chimeric receptor constructs; GM and HS: conception and design, financial support, data analysis, manuscript writing.

## Supplementary Material

Additional file 1 **FGF-2 promotes the self-renewal of both rodent and human NSCs**. A. Sample images of mouse, rat and human NSCs cultured in the presence of FGF-2 and/or other growth factors. Scale bar: 20 μm. B. Sample images of rat NSCs in various conditions. Scale bar: 20 μm. Click here for file

Additional file 2 **Clonal analysis assay**. A. A schematic diagram of clonal analysis assay. B. Sample images and quantification of the effect of FGF-2 on clonal expansion. Scale bar: 20 μm. Click here for file

Additional file 3 **Defective neuronal differentiation in PLCγ1-depleted cells**. Immunostaining of differentiated culture from adult NSCs with PLCγ1 depleted. Shown are Tuj1 (**A**), DAPI (**B**), ZsGreen (**C**) and Merged image (**D**). Scale bar: 20 μm. Click here for file

Additional file 4 **Model**. A model of the intracellular signalling mechanisms by which FGF-2 promotes the self-renewal of adult NSCs. Click here for file
