# Supplementary figures and images for: Molecular genetic analysis of FGFR1 signalling reveals distinct roles of MAPK and PLCγ1 activation for self-renewal of adult neural stem cells
Source: Mol Brain. 2009 Jun 8;2:16. doi: 10.1186/1756-6606-2-16 (PMC2700800; doi:10.1186/1756-6606-2-16)

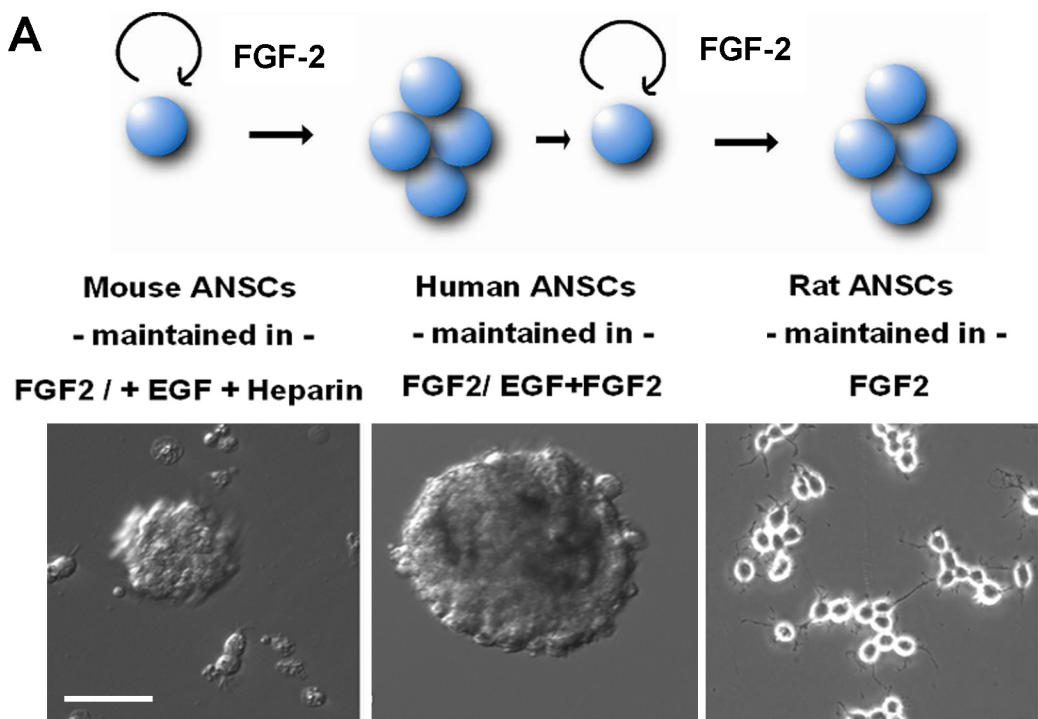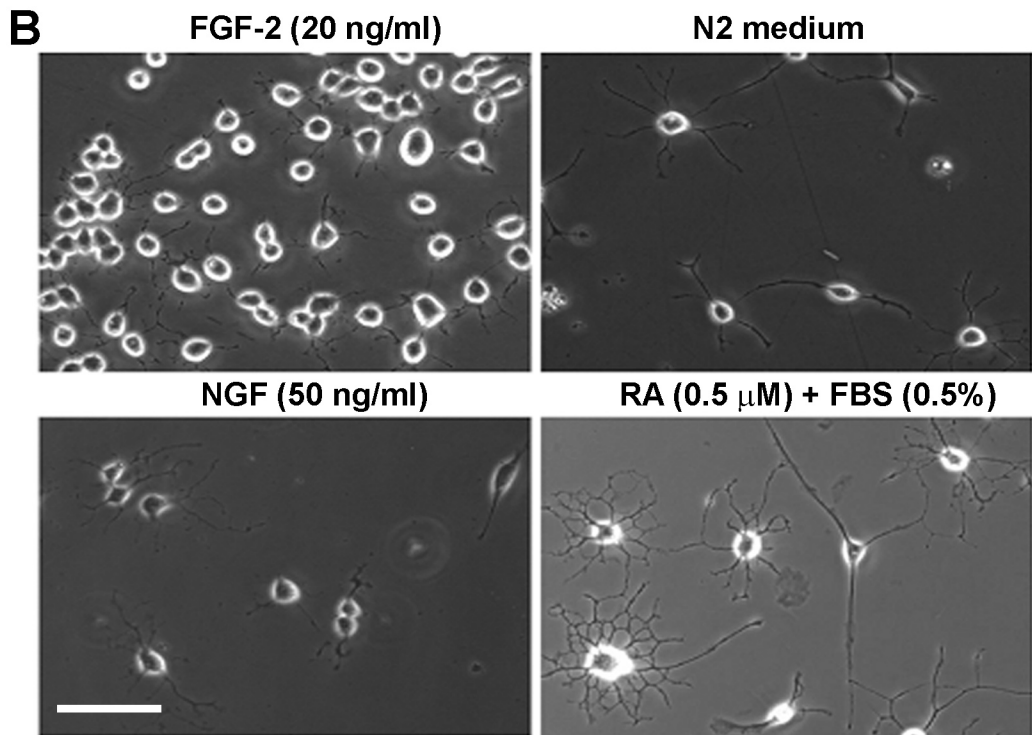

Supplement: Additional file 1 — FGF-2 promotes the self-renewal of both rodent and human NSCs. A. Sample images of mouse, rat and human NSCs cultured in the presence of FGF-2 and/or other growth factors. Scale bar: 20 μm. B. Sample images of rat NSCs in various conditions. Scale bar: 20 μm. [file 1756-6606-2-16-S1.pdf]

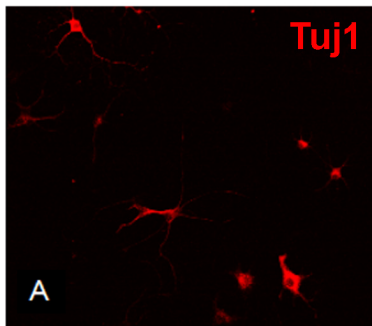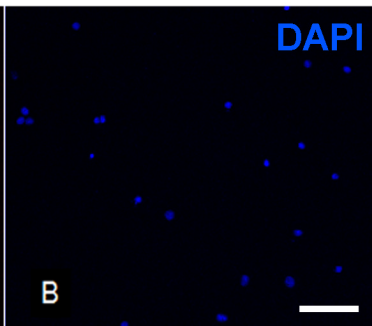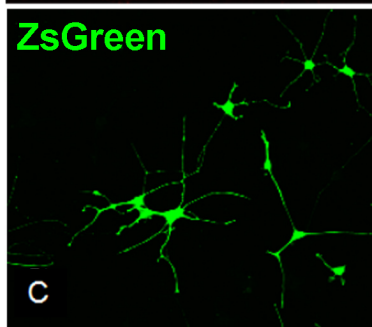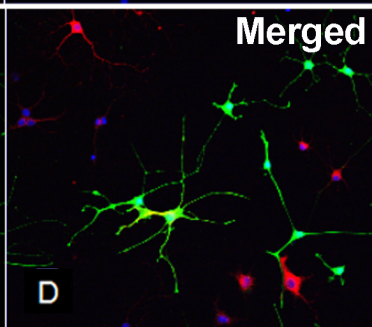

Supplement: Additional file 3 — Defective neuronal differentiation in PLCγ1-depleted cells. Immunostaining of differentiated culture from adult NSCs with PLCγ1 depleted. Shown are Tuj1 (A), DAPI (B), ZsGreen (C) and Merged image (D). Scale bar: 20 μm. [file 1756-6606-2-16-S3.pdf]
