# Supplementary material for: Molecular genetic analysis of FGFR1 signalling reveals distinct roles of MAPK and PLCγ1 activation for self-renewal of adult neural stem cells
Source: Mol Brain. 2009 Jun 8;2:16. doi: 10.1186/1756-6606-2-16 (PMC2700800; doi:10.1186/1756-6606-2-16)
Supplement: Additional file 2 — Clonal analysis assay. A. A schematic diagram of clonal analysis assay. B. Sample images and quantification of the effect of FGF-2 on clonal expansion. Scale bar: 20 μm. [file 1756-6606-2-16-S2.pdf]

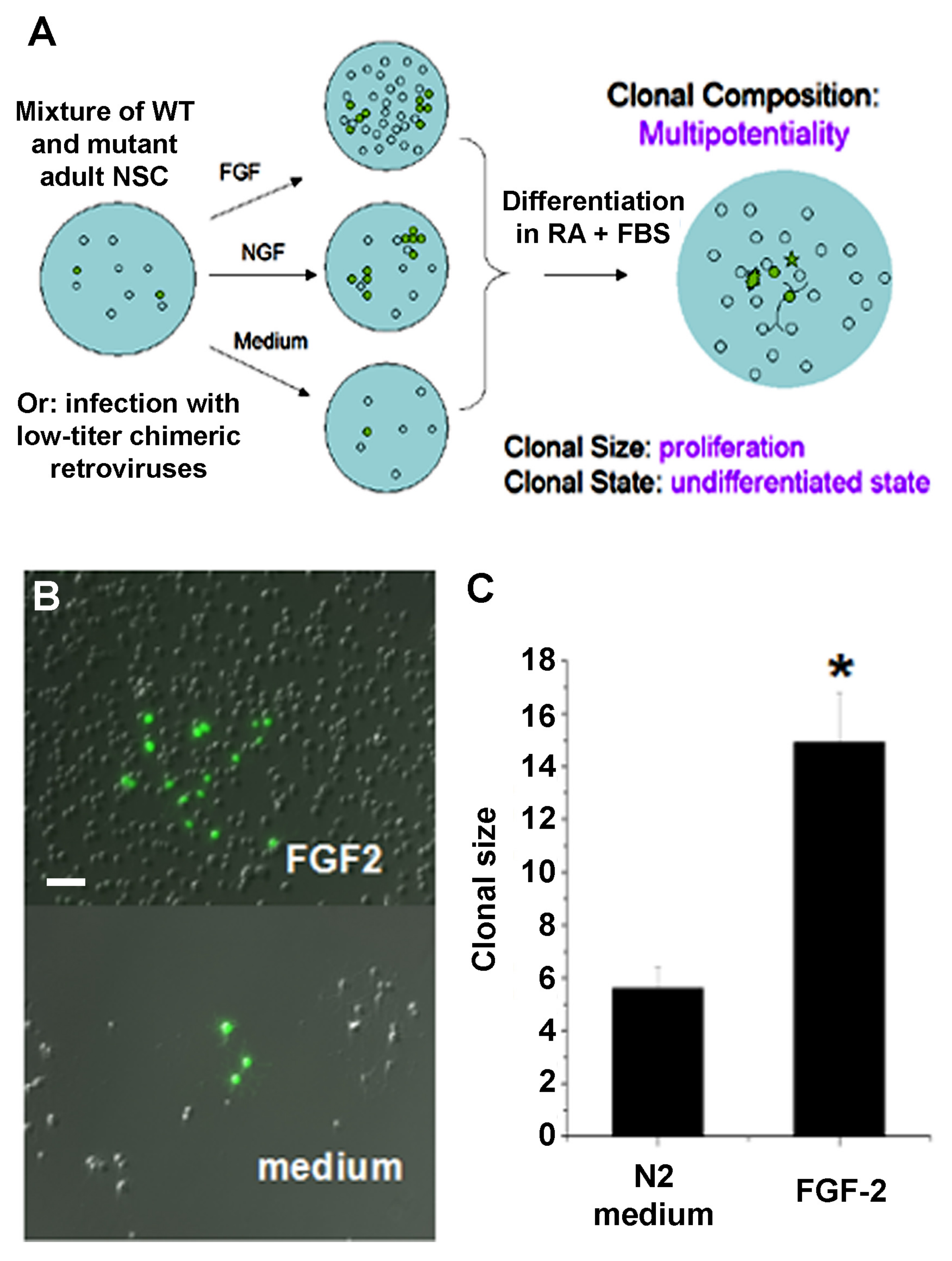

A
Mixture of WT and mutant adult NSC
FGF
NGF
Medium
Or: infection with low-titer chimeric retroviruses
Differentiation in RA + FBS
Clonal Composition: Multipotentiality
Clonal Size: proliferation
Clonal State: undifferentiated state
B
FGF2
medium
C
Clonal size
0
2
4
6
8
10
12
14
16
18
*
N2 medium
FGF-2
