# Supplementary material for: Molecular genetic analysis of FGFR1 signalling reveals distinct roles of MAPK and PLCγ1 activation for self-renewal of adult neural stem cells
Source: Mol Brain. 2009 Jun 8;2:16. doi: 10.1186/1756-6606-2-16 (PMC2700800; doi:10.1186/1756-6606-2-16)
Supplement: Additional file 4 — Model. A model of the intracellular signalling mechanisms by which FGF-2 promotes the self-renewal of adult NSCs. [file 1756-6606-2-16-S4.pdf]

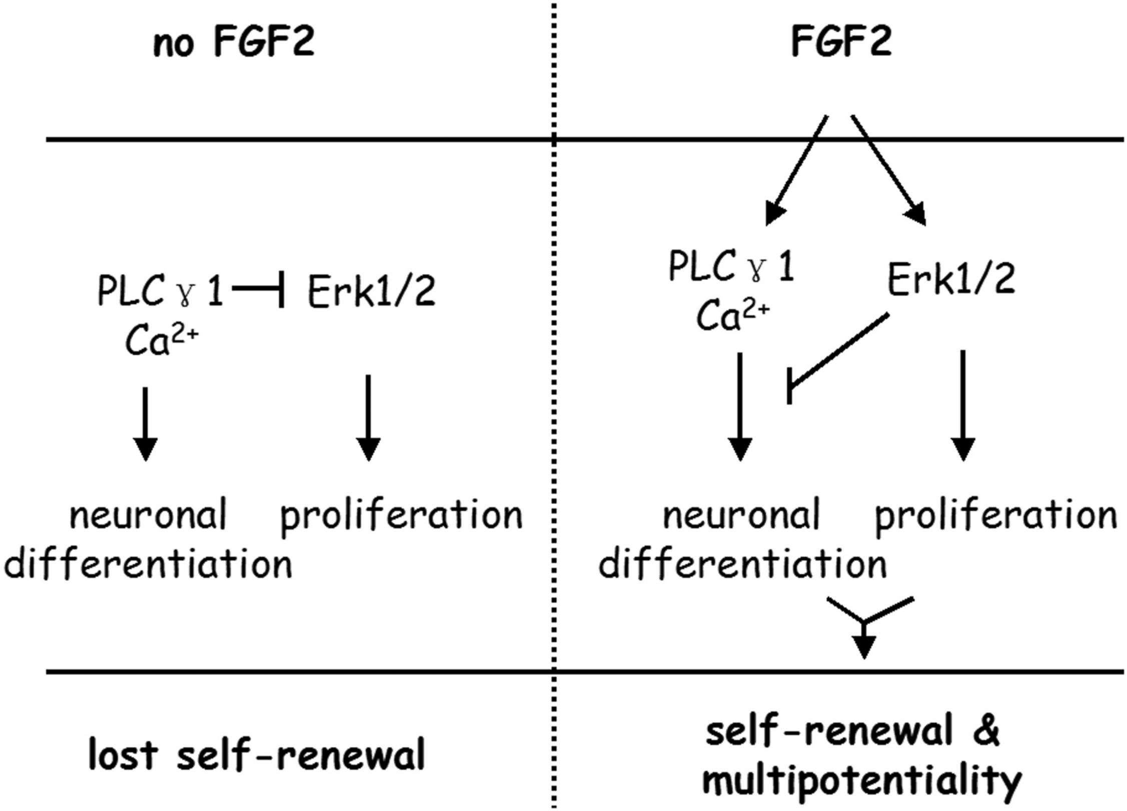

no FGF2
FGF2
PLCγ1
Ca2+
Erk1/2
PLCγ1
Ca2+
Erk1/2
neuronal differentiation
proliferation
neuronal differentiation
proliferation
lost self-renewal
self-renewal & multipotentiality
